# Supplementary material for: Estrogen-related receptor γ causes osteoarthritis by upregulating extracellular matrix-degrading enzymes
Source: Nat Commun. 2017 Dec 15;8:2133. doi: 10.1038/s41467-017-01868-8 (PMC5732273; doi:10.1038/s41467-017-01868-8)
Supplement: Supplementary file 1 — Supplementary Information [file 41467_2017_1868_MOESM1_ESM.pdf]

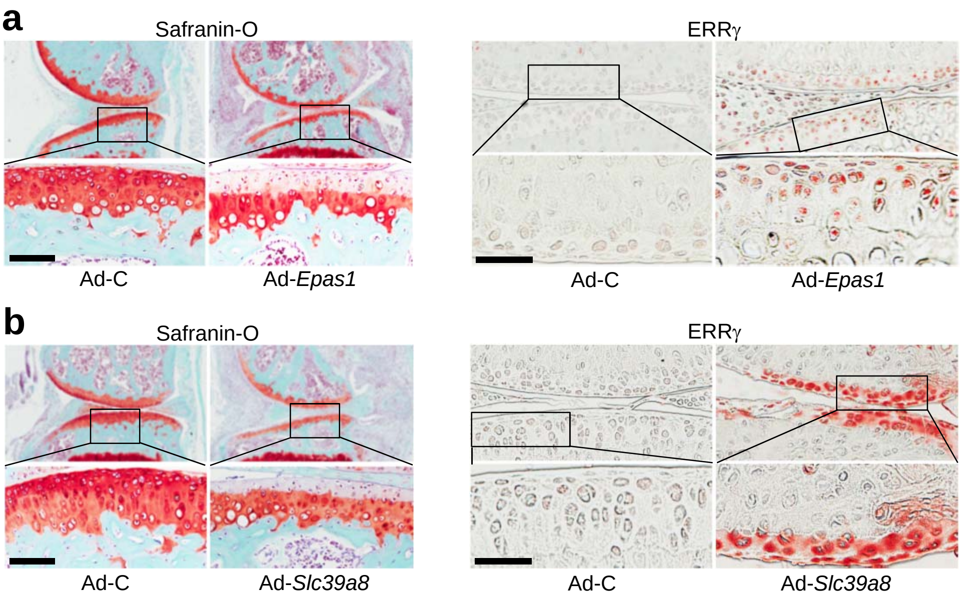

Supplementary Figure 1. ERRγ is upregulated in chondrocytes of OA cartilage from mouse models. (**a,b**) Safranin-O staining (left) and ERRγ immunostaining (right) in OA cartilage from mice subjected to HIF-2α overexpression via IA injection of Ad-*Epas1* (**a**) or adenovirus-mediated ZIP8 overexpression (**b**) (*n* = 5 mice per group). IA injection of Ad-C was used as a control.

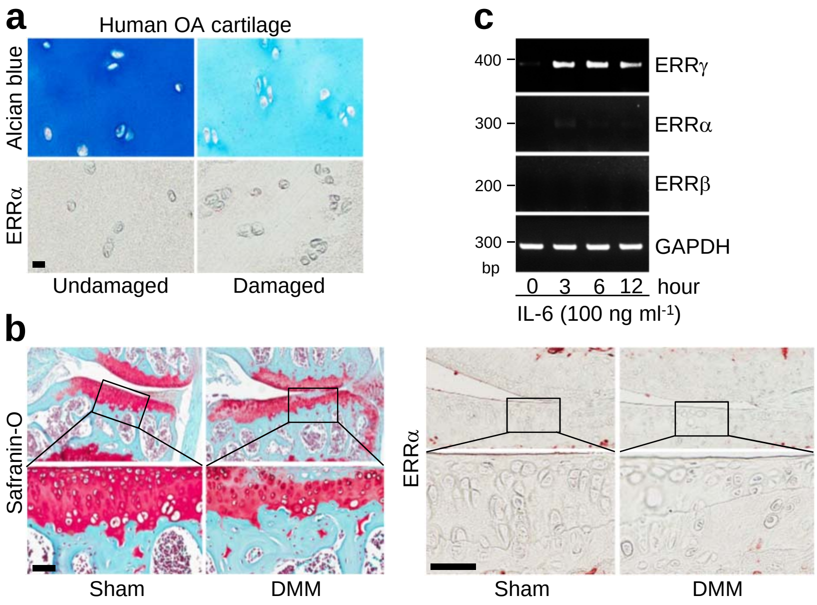

Supplementary Figure 2. ERRα is not detected in OA chondrocytes. (**a**) Representative images of alcian blue staining and ERRα immunostaining in damaged and undamaged regions of human OA cartilage (n = 6). (**b**) Representative images of safranin-O staining and ERRα immunostaining in cartilage sections of sham- and DMM-operated mice (n = 6 mice per group). (**c**) RT-PCR analysis of ERR isoforms in primary cultured mouse articular chondrocytes treated with IL-6 (n = 6). Scale bar: 50 μm.

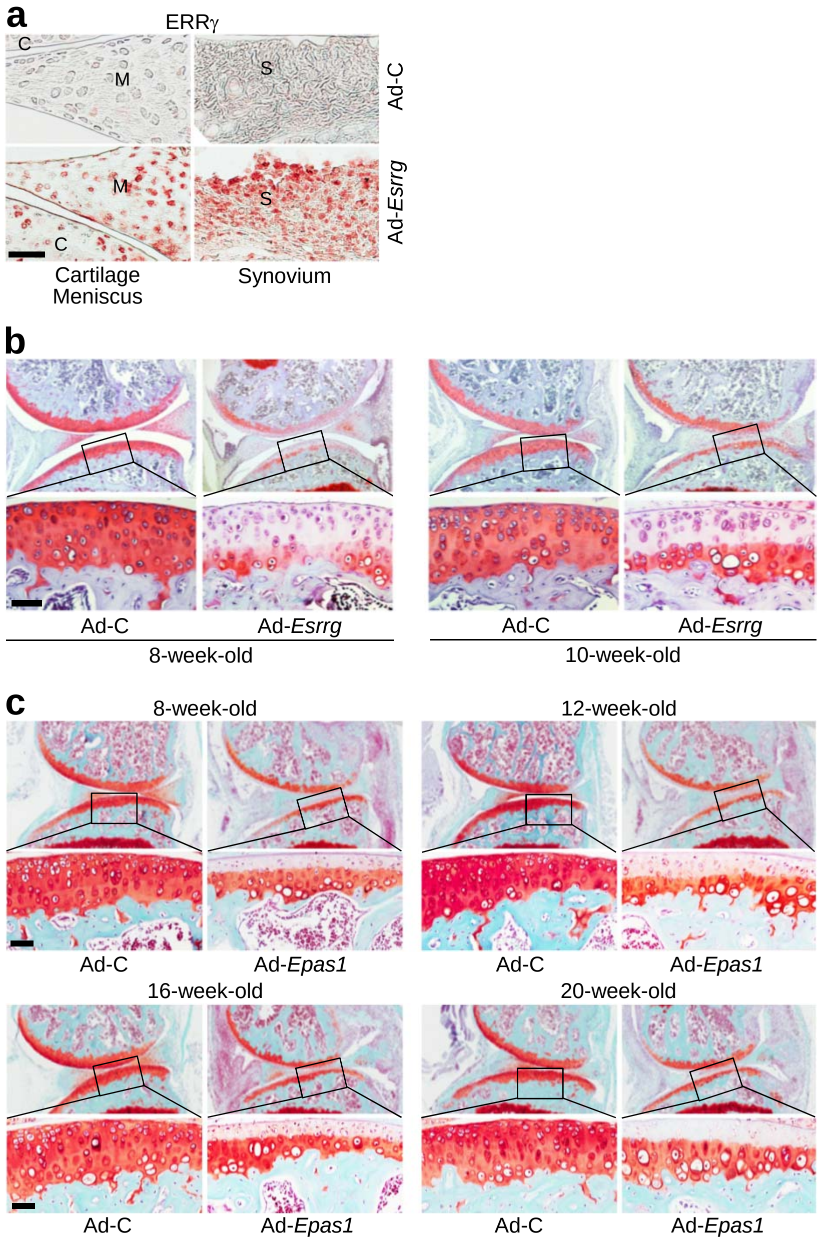

Supplementary Figure 3. Characterization of Ad-*Esrrg* IA injection. (**a**) Immunostaining for ERRγ in meniscus and synovial tissues of mice IA-injected with Ad-C (control) or Ad-*Esrrg* (to overexpress ERRγ) for 3 weeks. C, cartilage; M, meniscus; S, synovium. (**b**) Ad-C and Ad-*Esrrg* were IA-injected into 8- and 10-week-old mice. Safranin-O staining of joint sections, showing cartilage damage ($n \geq 8$ mice per group). (**c**) Mice of the indicated age were IA-injected with Ad-C or Ad-*Epas1*. Cartilage damage was detected by staining sections with safranin-O ($n = 6$ mice per group). Scale bar: 50 µm.

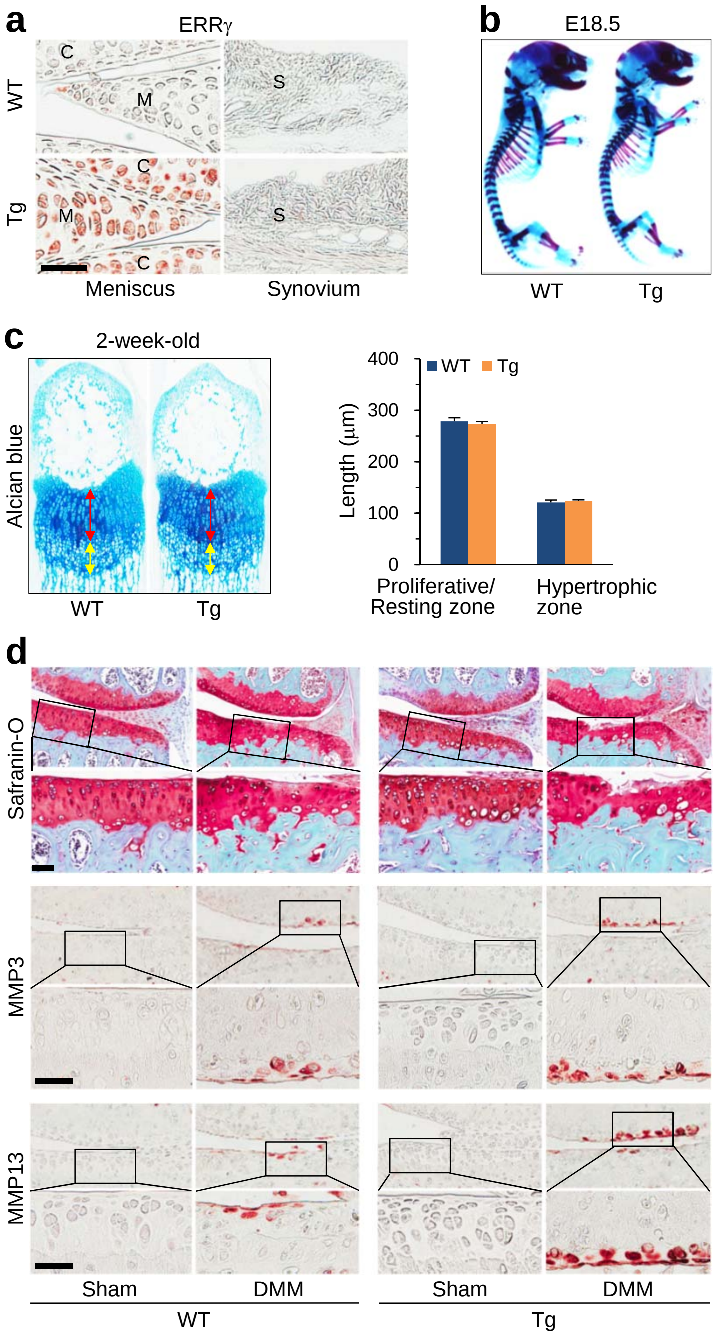

Supplementary Figure 4. Characterization of *Col2a1-Esrrg* Tg mice. (**a**) Immunostaining for ERRγ in meniscus and synovial sections of 10-week-old WT and Tg mice. C, cartilage; M, meniscus; S, synovium. (**b**) Representative skeletal staining images of E18.5 embryos of *Col2a1-Esrrg* Tg mice and WT littermates. (**c**) Alcian blue staining of 2-week-old metatarsal bones in Tg and WT littermates (left panel). Lengths of resting/proliferative and hypertrophic zones (right panel; $n \geq 10$ mice per group). (**d**) Tg mice and WT littermates ($n \geq 8$ mice per group) were subject to sham operation or DMM surgery. Cartilage sections were stained with safranin-O and immunostained for MMP3 and MMP13. Values are presented as means ± s.e.m. Scale bar: 50 µm.

a

Analysis of DMM surgery studies in recently published 196 papers (2013~2017)

Category	Sub-groups	Number of studies	
		No	%
Age	Juvenile (6-7 week)	3	1.5
	Young adult (8-11 week)	141	71.9
	Mature adult (12-39 week)	49	25.0
	Middle aged (40-71 week)	1	0.5
	Old (>72 week)	2	1.0
Duration of post-operation	Mild (1-3 week)	5	2.6
	Mild to moderate (4-7 week)	27	13.8
	Moderate to severe (8-12 week)	155	79.1
	Severe (>12 week)	9	4.6

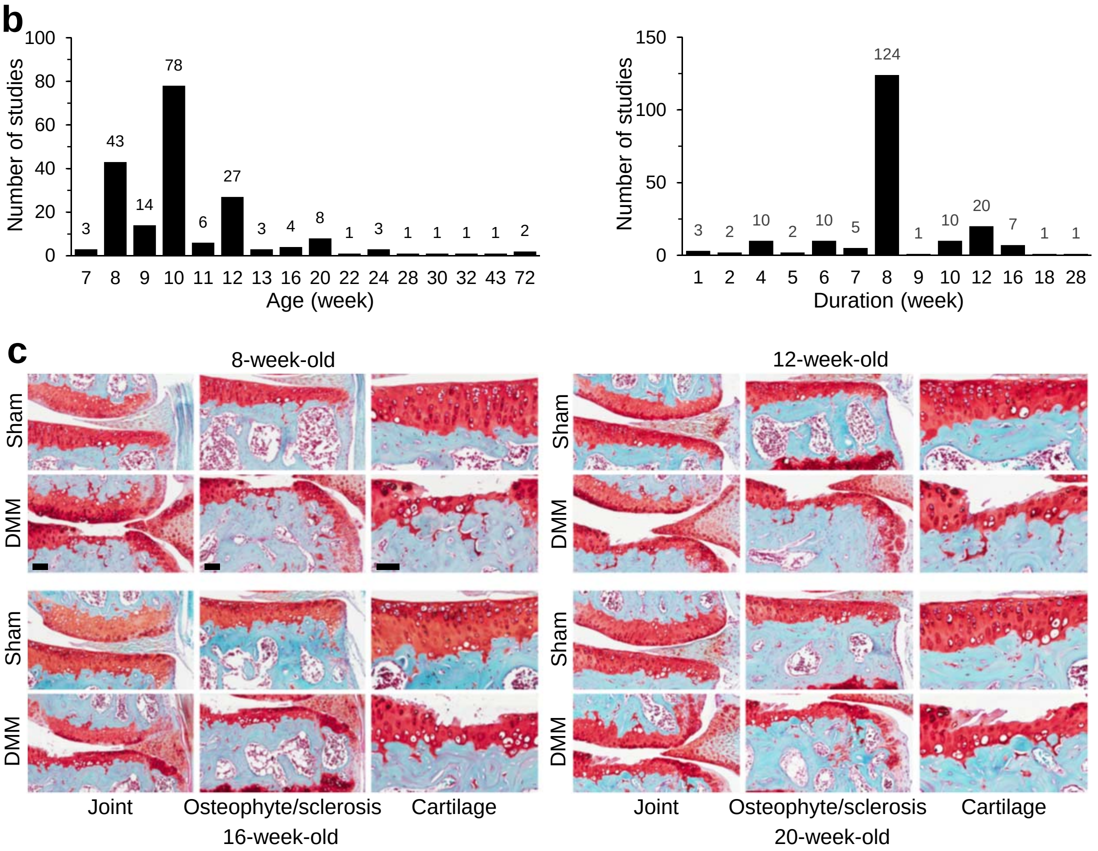

Supplementary Figure 5. Characterization of DMM surgery. (**a,b**) Analysis of DMM surgery studies in 196 recently published papers (2013–2017). (**c**) WT mice at the indicated ages were subjected to sham operation or DMM surgery. Representative images of safranin-O staining were obtained at 8 weeks after the operation (n = 15 mice per group). Scale bar: 50 μm.

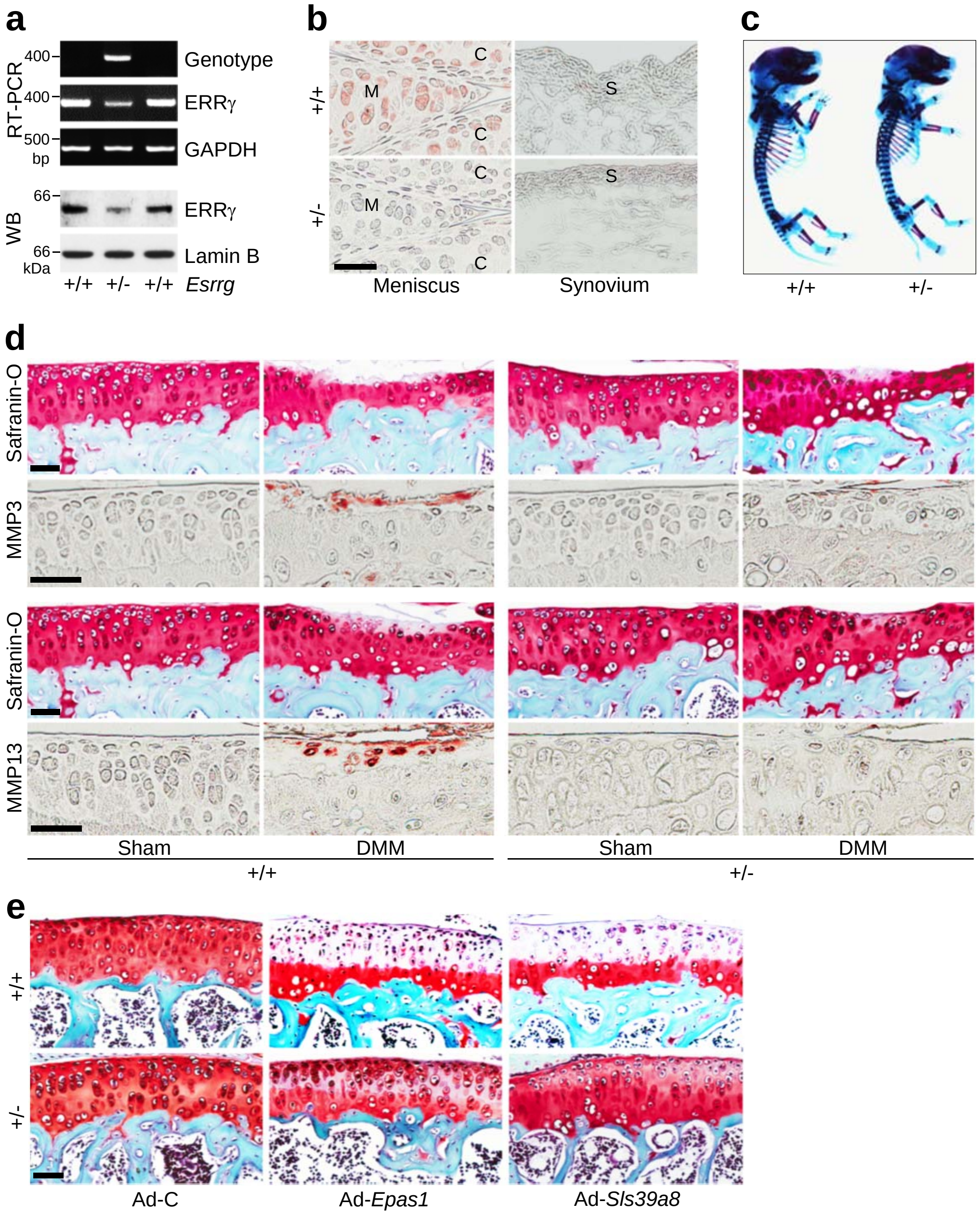

Supplementary Figure 6. Characterization of *Esrrg*$^{+/-}$ mice. (**a**) Genotypes of *Esrrg*$^{+/-}$ mice and WT (+/+) littermates. RT-PCR and Western blotting were used to examine mRNA and protein levels, respectively, of ERRγ in primary cultured chondrocytes isolated from *Esrrg*$^{+/-}$ and WT littermates. GAPDH and lamin B were used as loading controls. (**b**) Immunostaining for ERRγ proteins in joint tissues of *Esrrg*$^{+/-}$ and WT littermates. C, cartilage; M, meniscus; S, synovium. (**c**) Skeletal staining of E18.5 embryos of *Esrrg*$^{+/-}$ and WT mice. (**d**) *Esrrg*$^{+/-}$ and WT littermates were subject to sham operation or DMM surgery. Cartilage sections were stained with safranin-O and immunostained for MMP3 (upper) and MMP13 (lower). (**e**) *Esrrg*$^{+/-}$ and WT littermates were IA-injected with Ad-C, Ad-*Epas1*, or Ad-*Slc39a8*. Mice were sacrificed 3 weeks after the first injection, and cartilage sections were stained with safranin-O to detect cartilage destruction. Scale bar: 50 μm.

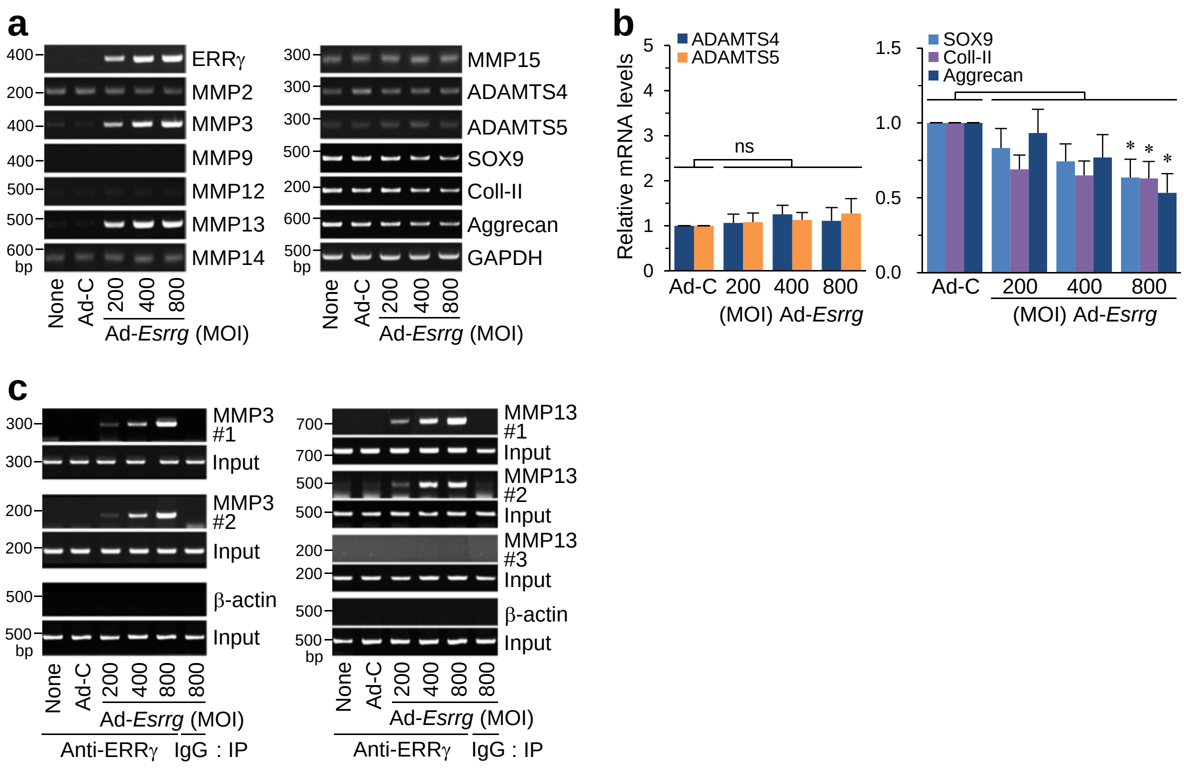

Supplementary Figure 7. ERRγ regulation of catabolic and anabolic factors in chondrocytes. (**a**) mRNA levels of the indicated anabolic and catabolic factors were analyzed in primary cultured chondrocytes infected with Ad-C (800 MOI) or the indicated MOI of Ad-*Esrrg* for 36 hours. (**b**) mRNA levels of the indicated molecules were quantified in chondrocytes infected with Ad-C (800 MOI) or the indicated MOI of Ad-*Esrrg* (n = 12). (**c**) Binding of ERRγ to the indicated ERRE sequences was determined by ChIP assay of chondrocytes infected with 800 MOI of Ad-C or the indicated MOI of Ad-*Esrrg*. β-Actin was used as a negative control, and IgG was used as an isotype control for the anti-ERRγ antibody ($n \geq 4$). Values are presented as means ± s.e.m. (*$P < 0.05$, **$P < 0.005$, and ***$P < 0.0005$; ns, not significant. One-way ANOVA).

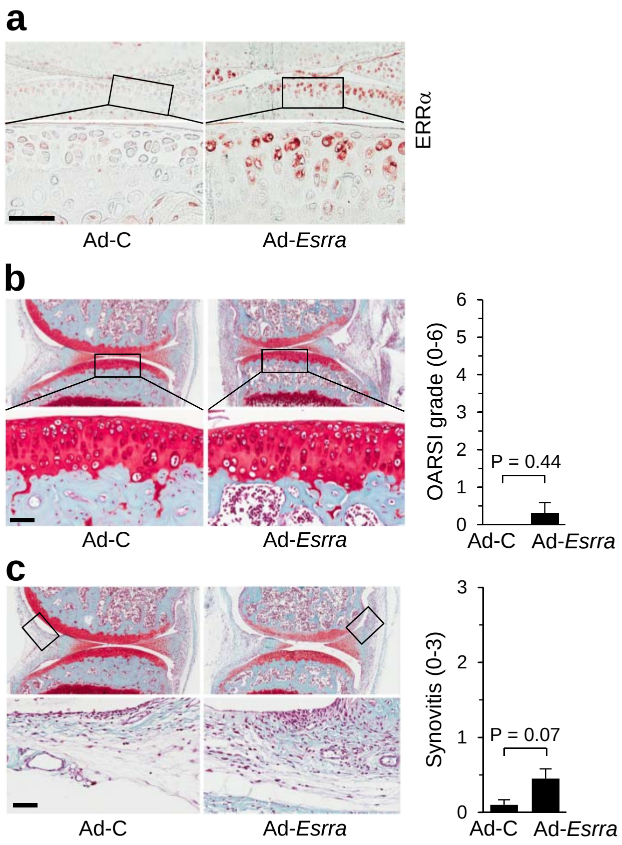

Supplementary Figure 8. Overexpression of ERRα does not cause cartilage damage or synovitis. (**a**) Immunostaining for ERRα in cartilage sections from mice IA-injected with Ad-C (control) or Ad-*Esrra* (to overexpress ERRα) for 3 weeks. (**b,c**) Ad-C and Ad-*Esrra* were IA-injected into 10-week-old mice. Safranin-O staining and OARSI grade (**b**; n = 6 mice per group) and H&E staining and synovial inflammation (**c**; n = 6 mice per group). Values are presented as means ± s.e.m. Mann-Whitney U test for OARSI grade and two-tailed t-test for synovitis. Scale bar: 50 µm.

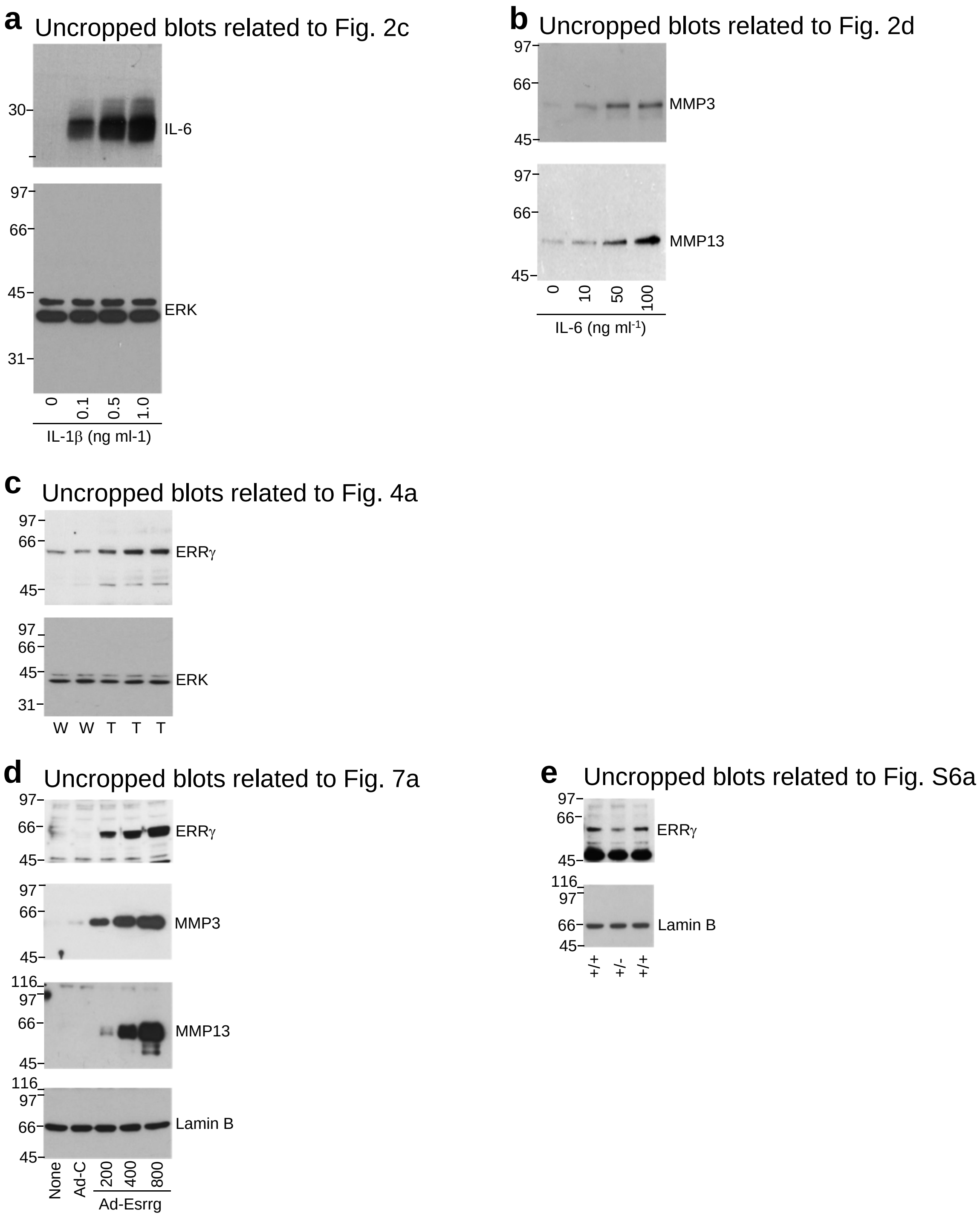

Supplementary Figure 9. Uncropped scans of the immunoblots. (**a**-**e**) Uncropped immunoblots related to Fig. 2c (**a**), 2d (**b**), 4a (**c**), 7a (**d**), and S6a (**e**).

Supplementary Table 1. Microarray analysis of the mRNA levels of ERRγ upstream signaling components in chondrocytes treated with IL-1β (36 hours, 1 ng ml^{-1}) or infected (36 hours) with 800 MOI of Ad-*Epas1* (to overexpress HIF-2α) or Ad-*Slc39a8* (to overexpress ZIP8).

Upstream signal	Gene symbol	Protein	Microarray (fold change)		
			IL-1β	HIF-2α	ZIP8
Alcohol	*Cnr1*	Cannabinoid receptor 1	-1.484 ± 0.120	-1.259 ± 0.008	-1.172 ± 0.109
	Mapk8	Mitogen-activated protein kinase 8	1.366 ± 0.131	1.099 ± 0.046	1.009 ± 0.088
	Jun	Jun proto-oncogene	2.359 ± 0.079	1.492 ± 0.154	1.090 ± 0.106
Inflammation	*Il6*	Interleukin 6	70.287 ± 0.291	5.321 ± 0.244	3.782 ± 0.305
	Il6ra	Interleukin 6 receptor, α	-1.026 ± 0.105	-1.167 ± 0.084	-1.075 ± 0.091
	Jak1	Janus kinase 1	1.511 ± 0.023	1.482 ± 0.069	1.263 ± 0.044
	Jak2	Janus kinase 2	-1.298 ± 0.105	1.078 ± 0.042	1.084 ± 0.044
	Jak3	Janus kinase 3	-1.078 ± 0.086	1.196 ± 0.114	1.186 ± 0.097
	Stat3	Signal transducer and activator of transcription 3	1.230 ± 0.030	1.397 ± 0.055	1.231 ± 0.058
Fasting	*Gcgr*	Glucagon receptor	1.027 ± 0.072	-1.002 ± 0.092	-1.071 ± 0.084
	Prkaca	Protein kinase A, α subunit	1.167 ± 0.063	1.233 ± 0.095	1.125 ± 0.089
	Creb1	cAMP responsive element binding protein 1	-1.071 ± 0.079	1.067 ± 0.048	-1.051 ± 0.044
	Creb2	cAMP responsive element binding protein 2	-	-	-
	Creb3	cAMP responsive element binding protein 3	-1.208 ± 0.076	-1.128 ± 0.050	-1.194 ± 0.036
Feeding	*Insr*	Insulin receptor	1.130 ± 0.104	-1.374 ± 0.098	-1.338 ± 0.042
	Akt	Thymoma viral proto-oncogene 1	-1.449 ± 0.048	1.019 ± 0.057	1.027 ± 0.049
	Sik2	Salt-inducible kinase 2	1.299 ± 0.082	1.051 ± 0.099	1.029 ± 0.097
	Crtc2	CREB regulated transcription coactivator 2	1.036 ± 0.039	1.064 ± 0.119	-1.007 ± 0.098
Hypoxia	*Hif1a*	Hypoxia-inducible factor 1, α subunit	1.202 ± 0.072	1.440 ± 0.043	1.260 ± 0.038
	Epas1	Hypoxia-inducible factor 2, α subunit	10.033 ± 0.139	58.574 ± 0.056	3.683 ± 0.135
ER stress	*Atf6*	Activating transcription factor 6	-1.187 ± 0.099	-1.333 ± 0.056	-1.222 ± 0.055
ERRs	*Esrra*	ERRα	1.03 ± 0.122	1.05 ± 0.043	0.99 ± 0.079
	Esrrb	ERRβ	1.06 ± 0.077	0.85 ± 0.176	1.01 ± 0.189
	Esrrg	ERRγ	0.87 ± 0.067	0.87 ± 0.091	0.91 ± 0.114

Supplementary Table 2. PCR primers and conditions.

Genes	Strand	Primer sequences	Size (bp)	AT (°C)	Origin
Adamts4	S<br>AS	5′-CATCCGAAACCCTGTCAACTTG-3′<br>5′-GCCCATCATCTTCCACAATAGC-3′	281	58	Mouse
Adamts5	S<br>AS	5′-GCCATTGTAATAACCCTGCACC-3′<br>5′-TCAGTCCCATCCGTAACCTTTG-3′	292	58	Mouse
Acan	S<br>AS	5′-CTGTCTTTGTCACCCACACATG-3′<br>5′-GAAGACGACATCACCATCCAG-3′	581	55	Mouse
Col2a1	S<br>AS	5′-CACACTGGTAAGTGGGGCAAGACCG-3′<br>5′-GGATTGTGTTGTTTCAGGGTTCGGG-3′	173	57	Mouse
Epas1	S<br>AS	5′-CGAGAAGAACGACGTGGTGTTC-3′<br>5′-GTGAAGGCTGGCAGGCTCC-3′	333	64	Mouse
Esrra	S<br>AS	5′-TGCCAATTCTGACTCTGTGC-3′<br>5′-ATCATGGCCTCAAGCATTC-3′	264	60	Mouse
ESRRA	S<br>AS	5′-TGCCAATTCAGACTCTGTGC-3′<br>5′-CCTCGAGCATCTCCAAGAAC-3′	257	60	Human
Esrrb	S<br>AS	5′-CTAGGGGTTGAGCAGGACAA-3′<br>5′-ATCTCCATCCAGGCACTCTG-3′	200	60	Mouse
ESRRB	S<br>AS	5′-TGTCAAGCCATGATGGAAAA-3′<br>5′-GGTGAGCCAGAGATGCTTTC-3′	182	60	Human
Esrrg	S<br>AS	5′-AAGATCGACACATTGATTCCAGC-3′<br>5′-GCTTCACATGATGCAACCCC-3′	350	64	Mouse
ESRRG	S<br>AS	5′-AAGATCGACACATTGATTCCAGC-3′<br>5′-GCTTCACATGATGCAACCCC-3′	350	64	Human
Gapdh	S<br>AS	5′-TCACTGCCACCCAGAAGAC-3′<br>5′-TGTAGGCCATGAGGTCCAC-3′	450	58	Mouse
Mmp2	S<br>AS	5′-CCAACTACGATGATGAC-3′<br>5′-ACCAGTGTCAGTATCAG-3′	233	60	Mouse
Mmp3	S<br>AS	5′-AGGGATGATGATGCTGGTATGG-3′<br>5′-CCATGTTCTCCAACTGCAAAGG-3′	434	58	Mouse
Mmp9	S<br>AS	5′-TGCACTGGGCTTAGATCATTCC-3′<br>5′-CGGTCCTTGAAGAAATGCAGAG-3′	450	58	Mouse
Mmp12	S<br>AS	5′-CCCAGAGGTCAAGATGGATG-3′<br>5′-GGCTCCATAGAGGGACTGAA-3′	482	60	Mouse
Mmp13	S<br>AS	5′-TGATGGACCTTCTGGTCTTCTGG-3′<br>5′-CATCCACATGGTTGGGAAGTTCT-3′	473	58	Mouse
Mmp14	S<br>AS	5′-GTGCCCTAGGCCTACATCCG-3′<br>5′-TTGGGTATCCATCCATCACT-3′	580	55	Mouse
Mmp15	S<br>AS	5′-GAGAGATGTTTGTGTTCAAGGG-3′<br>5′-TGTGTCAATGCGGTCATAGGG-3′	260	62	Mouse
Sox9	S<br>AS	5′-CACTGGCAGTTACGGCATCAG-3′<br>5′-CATGTAAGTGAAGGTGGAGTAGAGC-3′	457	61	Mouse
Slc36a8	S<br>AS	5′-GAACAATTGCCTGGATGATCACGC-3′<br>5′-AAGCCGGTTAACATCCCTGCATTC-3′	430	58	Mouse

AT, annealing temperature; S, sense; AS, antisense

Supplementary Table 3. PCR primers and conditions for ChIP assays.

Target	Strand	Primer sequences	Size (bp)	AT (°C)	Origin
Mmp3 #1(F)	S<br>AS	5´-CACCTCCCCCTGTCATTTAG-3´<br>5´-GGGATCAAACTCAAGCCATC-3´	300	59	Mouse
Mmp3 #2(F)	S<br>AS	5´-AGCCACTACCAGATCCTTGC-3´<br>5´-GAGGGAGCAAAGGGAAAAAC-3´	198	60	Mouse
Mmp13 #1(F1)(F2)	S<br>AS	5´-TTGACCATGGGGCTAGAAAG-3´<br>5´-GCCTCAGCATTTTCATGGAT-3´	769	60	Mouse
Mmp13 #2(R1)(R2)	S<br>AS	5´-TGCCACATCACCTCCAATAA-3´<br>5´-CTTTCTAGCCCCATGGTCAA-3´	500	60	Mouse
Mmp13 #3(F)	S<br>AS	5´-GTGGCACAATGGTTTGAGTG-3´<br>5´-CCATCACTCCCAGTCAGGTT-3´	181	60	Mouse
Mmp13 #4(R)	S<br>AS	5´-CATAAGGCCAACCTCAGCTC-3´<br>5´-TGGCGTTTGAAACTGTTCTG-3´	568	60	Mouse

AT, annealing temperature; S, sense; AS, antisense
